# Nanocomposite films based on chia (*Salvia hispanica* L.) flour seeds incorporating antioxidant chitosan nanoparticles

**DOI:** 10.3389/fchem.2024.1448171

**Published:** 2024-08-07

**Authors:** Gema Morales-Olán, Pedro Moreno-Zarate, María Antonieta Ríos-Corripio, Aleida Selene Hernández-Cázares, Marlon Rojas-López, Silvia Luna-Suárez

**Affiliations:** ^1^ Colegio de Postgraduados Campus Córdoba, Veracruz, Mexico; ^2^ Instituto Nacional de Astrofísica, Óptica y Electrónica, Puebla, Mexico; ^3^ CONAHCYT-Colegio de Postgraduados Campus Córdoba, Veracruz, Mexico; ^4^ Instituto Politécnico Nacional, Centro de Investigación en Biotecnología Aplicada, Tepetitla, Tlaxcala, Mexico

**Keywords:** biodegradable films, sustainable packaging, food preservation, antioxidant activity, active packaging

## Abstract

Chia (*Salvia hispanica* L.) flour seeds produce films with good barrier properties against water vapor and could be used as food packaging; however, their mechanical properties are poor, which limits their application. The incorporation of nanoparticles into natural polymers is a strategy used to improve the properties of films to increase their applications. Furthermore, nanoparticles can encapsulate antioxidant agents and generate active films. The objective of this study was to evaluate the influence of chia flour (4%–7%), glycerol (15%–25%), and chia extract-loaded chitosan nanoparticles (ChCNp) (0%–0.75%) on the physical, mechanical, barrier, structural and antioxidant properties of chia flour nanocomposite films. Chitosan nanoparticles loaded with antioxidant chia extract were synthesized by ionic gelation and incorporated into the films. The thickness, water vapor permeability, tensile strength, and antioxidant properties of the films were evaluated using a Box-Behnken experimental design. Structural analysis was conducted using the FTIR technique. The results of the ANOVA of the responses were adjusted to second and third order polynomial models obtaining determination coefficients of 0.96–0.99. The water vapor permeability of the films was 3.89 × 10^-8^–1.68 × 10^−7^ g mm/Pa s m^2^, tensile strength was 0.67–3.59 MPa and antioxidant activity was 57.12%–67.84%. The variables presented different effects on the films. Increasing the chia seed flour concentration negatively affected the water vapor permeability but improved the tensile strength and the antioxidant capacity of the films. The increase in glycerol concentration caused the films to become brittle. The nanoparticles had a significant effect on the thickness of the films and improved their mechanical and antioxidant properties. However, they did not show an effect on barrier properties. The results demonstrate that it is possible to obtain nanocomposite films with antioxidant capacity from chia seed flour and with the incorporation of chitosan nanoparticles loaded with antioxidants.

## 1 Introduction

The interest in developing packaging films based on biopolymers and natural bioactive substances has increased recently (Rao et al., 2023). Films with antioxidant and antimicrobial functional properties help to extend the shelf life of food, reducing microbial growth and preventing spoilage reactions. They are also biodegradable, avoiding the environmental pollution caused by synthetic packaging. Chia (*Salvia hispanica* L.) is a native plant from Mexico and Guatemala. Its seeds contain biopolymers such as proteins (15%–25%), carbohydrates (25%–41%), dietary fibre (34%), and lipids (30%–33%) (Vera-Cespedes et al., 2023). These polymers can be used to develop films. The film-forming properties of chia mucilage have been widely studied (Muñoz et al., 2012; Dick et al., 2015a; Salazar et al., 2020; Urbizo-Reyes et al., 2020; Kosarsoy, 2022; Semwal et al., 2022). However, there are few studies on films made from chia seed flour. Dick et al. (2015b) evaluated the physicochemical, structural, barrier, and mechanical properties of chia flour-based films, reporting that the films presented excellent barrier properties but inferior mechanical properties, so their application is reduced. A strategy applied to improve the mechanical properties of films is the incorporation of nanoparticles. The nanostructures can interact with the polymeric matrix, thus increasing their resistance (Dash et al., 2019). Mujtaba et al. (2019a) incorporated cellulose nanofibers into chia mucilage films, and the particles improved the tensile strength. The addition of starch nanocrystals has also been reported; however, these particles did not significantly affect the mechanical properties of chia mucilage films (Mujtaba et al., 2019b). Not all nanoparticles interact in the same way with polymeric matrices, which is why it is not possible to generalize their action, and it is important to evaluate their effect in each of the possible polymeric matrices. Chitosan is a biocompatible, non-toxic, antimicrobial polymer derived from chitin that can form nanoparticles by ionic gelation (Ju et al., 2020). The incorporation of chitosan particles into films improves their barrier and mechanical properties, as reported by various authors (Garavand et al., 2020; Lee et al., 2021). The effect of adding chitosan particles in flour films of chia seeds has been little studied. Chitosan nanoparticles can also serve as a vehicle for the incorporation of bioactive substances and generate nanocomposite active packaging (Roy and Rhim, 2021). Active packaging with natural antioxidant compounds is a novel alternative applied to prevent lipid oxidation in foods. These reactions together with microbial growth are the main causes of the loss of sensory and nutritional quality of foods (Abiodun et al., 2023). Due to the growing demand for natural antioxidants in the food industry, new sources are being sought for the extraction of these compounds. In chia seeds, important phenolic compounds, such as kaempferol, quercetin, myricetin, and chlorogenic and caffeic acids have been identified (Morales-Olán et al., 2020) and the antioxidant capacity of the aqueous and ethanolic extracts of its seeds has been determined (Alcântara et al., 2019; Kulczyński et al., 2019). These extracts could be applied as antioxidant agents in the food industry. In previous studies, it was determined that the encapsulation of the antioxidant ethanolic extract of chia seeds in chitosan particles is possible, with good encapsulation efficiency (Morales-Olán et al., 2021). These nanoparticles could be added to chia flour films to improve their mechanical properties and obtain active nanocomposite packaging films. The objective of this study was to evaluate the effect of the content of chia flour (4%–7%), glycerol (15%–25%), and chia extract-loaded chitosan nanoparticles (ChCNp) (0%–0.75%) on the physical, barrier, mechanical, structural and antioxidant properties of chia flour nanocomposite films.

## 2 Materials and methods

### 2.1 Materials and reagents

Chia seeds were acquired from local producers in Puebla City, Mexico. Seeds were ground in a food processor (Krups GX410011, Groupe SEB, Solingen, Germany), and the particle size was homogenized by passage through a 60-mesh screen to generate the flour. Sodium tripolyphosphate (TPP), chitosan (75%–85% deacetylated, medium molecular mass), glacial acetic acid (≥99.5%), glycerol, ethanol (≥99.5%) and 2,2-diphenyl-1-picrylhydrazyl (DPPH) were obtained from Sigma-Aldrich (Sigma Co., Saint Louis, United States).

### 2.2 Synthesis and characterization of chia extract-loaded chitosan nanoparticles

The ChCNp were prepared following the methodology proposed by Morales-Olán et al. (2021). Briefly, chia extract (0.2 mg/mL) was mixed with the TPP (0.07%, w/v). The solution was slowly added to the chitosan solution (0.05%) with stirring at 25°C. Chitosan particles with chia extract were collected by centrifugation (MultifugeTM X3; Thermo ScientificTM) at 12,000 rpm for 30 min. Characterization of the particles included the determination of the encapsulation efficiency (EE), loading capacity (LC), size, morphology, and zeta potential. The size and morphology of the particles were examined by using a Scanning Electron Microscope (SEM-FE-JOL 7610F, JEOL, Tokyo, Japan) with a secondary electron detector and an Oxford EBSD detector (2.0 KV). The images were analyzed with ImageJ 1.52a to determine the particle size. The zeta potential (ζ) was measured using a Zetasizer Nano ZSP (Malvern Instruments, United Kingdom). Measurements were made in triplicate at 25°C. The EE and LC were calculated using Equations 1, 2.

$$EE\;\left(\%\right)=\left(mass\;of\;loaded\;extract/mass\;of\;initial\;extract\right)\;x\;100$$ (1)

$$LC\;\left(\%\right)=\left(mass\;of\;loaded\;extract/mass\;of\;sample\right)\;x\;100$$ (2)

### 2.3 Preparation of nanocomposite films

Chia flour (4%–7%, w/v) was dissolved in distilled water, the pH was adjusted to 10 and the solution was stirred for 25 min. The solutions were heated at 80°C for 15 min, followed by the addition of glycerol (15%–25%, dry basis). The mixture was centrifuged at 5,000 rpm for 10 min. The ChCNp (0%, 0.375%, and 0.75%, based on the weight of chia flour) were added to the film-forming solution and magnetically stirred for 10 min at 25°C, placed in silicone molds (12.57 cm^2^) and dried for 12 h at 40°C. Before characterization, the films were conditioned with saturated NaBr solutions (58% RH) for 48 h.

### 2.4 Film characterization

#### 2.4.1 Structural characterization of the films by FTIR

The nanoparticle-free and nanocomposite films were characterized by FTIR spectroscopy (Vertex 70v; Bruker, Bremen, Germany) with an attenuated total reflectance (ATR) accessory. The measurement was performed in triplicate in the region of 4,000 to 500 cm^−1^.

#### 2.4.2 Physical properties: thickness (T)

The thickness of the films was measured with a micrometer (MCD-1” MX, 0-1 IN, Mitutoyo, Kawasaki, Japan). The measurements were made in different areas of the films. The mean of the measurements was used to determine the mechanical and barrier properties.

#### 2.4.3 Barrier properties: water vapor permeability (WVP)

The cup method of the ASTM E96-95 standard was applied following the methodology of Beristain-Bauza et al. (2016) with slight modifications. The films were placed on top of beakers (25 mm diameter) with anhydrous silica (0% RH). The glasses were placed in a desiccator with NaCl solution (75% RH) at 25°C.

The water content transferred to the films was determined from the mass gain every 24 h for 120 h. WVP (g mm/Pa s m^2^) was calculated using Equation 3:

$$WVP=\left(WVT*L\right)/S\left(R_{1}-R_{2}\right)\;$$ (3)

Where, WVT = water vapor transmission (g/Pa s m^2^), calculated from the slope of the linear regression of the mass gain versus time divided by beakers’ area (A, m^2^); L = film’s thickness; S = saturation vapor pressure at 25°C (Pa); R_1_ = RH of saturated NaCl solution; R_2_ = RH of desiccant.

#### 2.4.4 Mechanical properties: tensile strength (TS)

A texture analyzer (EZS 500 N, Shimadzu, Kyoto, Japan) was used to measure the TS according to ASTM D882-18. The films were cut into 2.0 × 5.0 cm strips. The grip spacing was 45 mm and the crosshead speed was 0.5 mm/s.

#### 2.4.5 Antioxidant properties

The DPPH radical-scavenging activity was measured following the method reported by Chaichi et al. (2023) with brief modifications. The films were mixed with DPPH methanol solution (100 mM) for 30 min in the dark. The activity was determined with Equation 4, where A = Absorbance.

$$DPPH\;scavenging\;activity=\left(A_{control}-A_{sample}/A_{control}\right)x100$$ (4)

### 2.5 Experimental design and statistical analysis

A Box-Behnken experimental design (BBD) was applied to evaluate the influence of variables on the nanocomposite films. The independent variables were the chia flour content ( $X_{1}$ ), glycerol content ( $X_{2}$ ) and ChCNp content ( $X_{3}$ ). The code levels were chia flour: 4% (−1), 5.5% (0), 7% (+1); glycerol: 15% (−1), 20% (0), 25% (+1); ChCNp: 0% (−1), 0.375% (0), 0.75% (+1). The levels were selected based on preliminary experiments. The BBD statistical design was composed of 15 experimental trials (12 factorial points and three central points). The experimental results were processed with the Design Expert^®^ software (Statease, Minneapolis, United States). For the adjustment of the response as a function of the independent variables, the following second and third order polynomial equations were used, Equations 5, 6:

$$Y_{k}=\beta_{0}+\beta_{1}X_{1}+\beta_{2}X_{2}+\beta_{3}X_{3}+\beta_{11}X_{1}^{2}+\beta_{22}X_{2}^{2}+\beta_{33}X_{3}^{2}+\beta_{12}X_{1}X_{2}+\beta_{13}X_{1}X_{3}+\beta_{23}X_{2}X_{3\;}+\varepsilon$$ (5)

$$\begin{array}{l} Y_{k}=\beta_{0}+\beta_{1}X_{1}+\beta_{2}X_{2}+\beta_{3}X_{3}+\beta_{11}X_{1}^{2}+\beta_{22}X_{2}^{2}+\beta_{33}X_{3}^{2}+\beta_{12}X_{1}X_{2}+\beta_{13}X_{1}X_{3}+\beta_{23}X_{2}X_{3\;}+\beta_{12}X_{1}^{2}X_{2}\\ +\beta_{13}X_{1}^{2}X_{3}+\beta_{12}X_{1}X_{2}^{2}+\beta_{13}X_{1}X_{3}^{2}+\beta_{23}X_{2}^{2}X_{3}{+\beta }_{23}X_{2}X_{3}^{2}+\beta_{11}X_{1}^{3}+\beta_{22}X_{2}^{3}+\beta_{33}X_{3}^{3}+\varepsilon \end{array}$$ (6)

Where Y_k_ = response variables: T (mm), WVP (g mm/Pa s m^2^), TS (MPa), and DPPH radical-scavenging activity (%). Independent coded variables include the content of $X_{1}$ = chia flour (%), $X_{2}$ = glycerol (%) and $X_{3}$ = ChCNp (%). The properties of the films were evaluated in triplicate, and the mean and standard deviation (SD) were obtained. Analysis of variance (ANOVA) was used to determine the independent variables with significant effects on the responses.

## 3 Results and discussion

### 3.1 Characterization of chia extract-loaded chitosan nanoparticles

**FIGURE 1:**
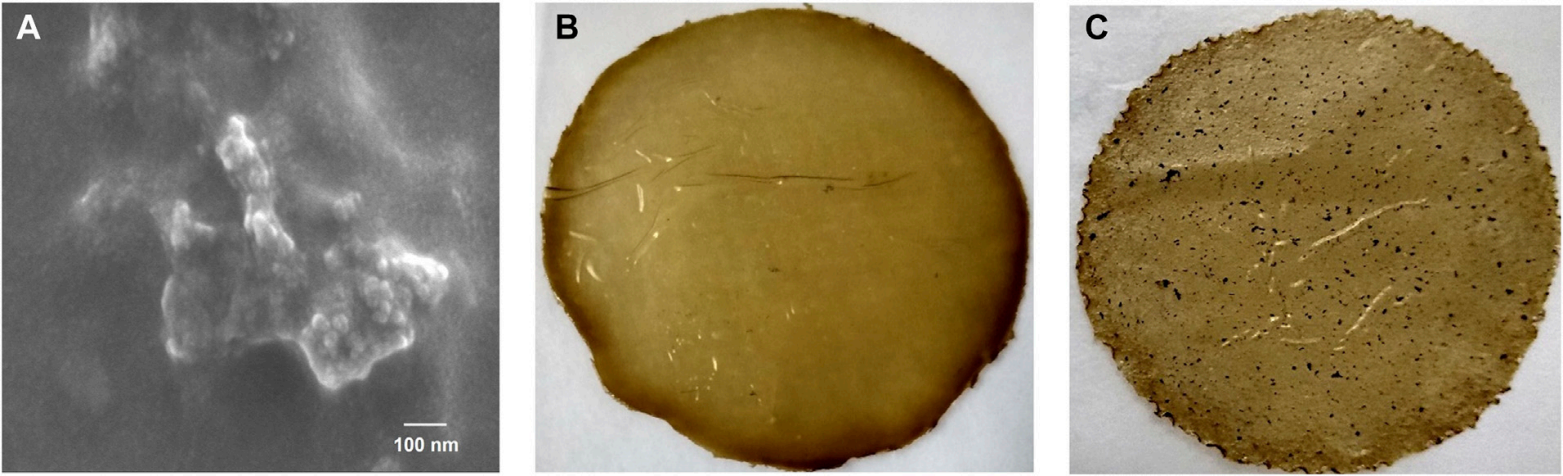
**(A)** SEM micrograph of chia extract loaded nanoparticles; **(B)** chia flour films without nanoparticles; **(C)** nanocomposite chia flour films with chia extract-loaded chitosan nanoparticles.

The nanoparticles presented an EE of 93.0% ± 4.5%. The EE was higher than that reported in the encapsulation of other antioxidant extracts, such as aqueous grape extract loaded in chitosan particles (51.90% ± 1.33%) (Soleymanfallah et al., 2022). The concentration of the extract used in the encapsulation of the particles was determined according to the results obtained in previous studies (Morales-Olán et al., 2021). The highest encapsulation efficiency was achieved with this concentration. The LC of the particles was 16.2% ± 1.5%. The values found were higher than those reported in chitosan particles loaded with resveratrol and tannic acid (Wu et al., 2017; Leonida et al., 2023). The particles presented a spherical morphology and a size of 39.7 ± 8.4 (Figure 1A). The zeta potential was 26.3 mV, this value showed that the synthesized colloidal particles were unstable and tended to agglomerate. The results agree with those reported by Ivancica et al. (2020).

### 3.2 Statistical analysis and model fitting

The results of the BBD employed to analyze the effect of the independent variables were as follows: the effects of chia flour (4%–7%), glycerol (15%–25%) and ChCNp (0.0%–0.75%) contents on the physical, mechanical, barrier, and antioxidant properties of chia films are shown in Table 1. The average measurements of the dependent variables were introduced into the design to obtain the fitting parameters and ANOVA (Table 2). The results showed that the quadratic regression models of the T and TS responses were statistically significant (*p* < 0.05). The lack of fit for the F values of the thickness and TS responses implies that the fit is not significant in relation to the pure error. There is a 9.9% and 5.9%, respectively, chance of lack of adjustment of the F value due to noise. The insignificance of the lack of fit indicates that the model fits the experimental data. All response variables presented a high R^2^ (Table 2), which shows a good correlation between the predicted and experimental values. Based on the results, these models are suitable to predict the responses in films made with chia flour containing chitosan particles encapsulating chia extract. The regression analysis for model fitting to assess the significance of the coefficient terms for all responses in the experimental design is shown in Table 3. For the T of the films, the regression coefficients for the factor chia flour ( $X_{1}$ ), glycerol ( $X_{2}$ ) and ChCNp ( $X_{3}$ ) were significant (*p < 0.05*). The WVP was significantly affected by the chia flour ( $X_{1}$ ). In the TS, the coefficients for chia flour ( $X_{1}$ ), glycerol ( $X_{2}$ ) and the interactions between flour and glycerol ( $\left.X_{1}X_{2}\right)$ and flour and nanoparticles ( $\left.X_{1}X_{3\;}\right)$ showed significance. The antioxidant activity was significantly affected by the chia flour ( $X_{1}$ ) and the interactions between flour and nanoparticles ( $\left.X_{1}X_{3}\right)$ and glycerol and nanoparticles ( $\left.X_{2}X_{3\;}\right)$ .

**TABLE 1:** Responses of the parameters used in the experimental design.

Runs	Independent variables/Coded variables			Dependent variables (responses) Experimental results			
	Chia flour (%), $x_{1}$	Glycerol (%), $x_{2}$	ChCNp (%) $x_{3}$	T (mm)	WVP (g mm/Pa s m^2^)	TS (MPa)	DPPH scavenging activity (%)
1	4 (−1)	15 (−1)	0.375 (0)	0.02 ± 0.00^a^	3.89 × 10^−8^ ± 0.28^a^	1.07 ± 0.27^a^	60.41 ± 0.48^a^
2	7 (1)	15 (−1)	0.375 (0)	0.03 ± 0.01^b^	9.46 × 10^−8^ ± 0.26^a^	3.59 ± 0.67^b^	60.69 ± 0.00^a^
3	4 (−1)	25 (1)	0.375 (0)	0.02 ± 0.00^a^	5.31 × 10^−8^ ± 0.39^a^	0.72 ± 0.00^a^	61.13 ± 0.71^a^
4	7 (1)	25 (1)	0.375 (0)	0.03 ± 0.01^b^	1.68 × 10^−7^ ± 0.07^a^	0.67 ± 0.20^a^	61.03 ± 0.49^a^
5	4 (−1)	20 (0)	0.000 (−1)	0.02 ± 0.00^a^	6.10 × 10^−8^ ± 0.07^a^	1.30 ± 0.57^a^	65.04 ± 0.49^a^
6	7 (1)	20 (0)	0.000 (−1)	0.04 ± 0.00^c^	1.17 × 10^−7^ ± 0.04^a^	1.48 ± 0.14^a^	57.12 ± 0.73^a^
7	4 (−1)	20 (0)	0.750 (1)	0.02 ± 0.00^a^	6.47 × 10^−8^ ± 0.00^a^	0.72 ± 0.28^a^	59.37 ± 0.71^a^
8	7 (1)	20 (0)	0.750 (1)	0.03 ± 0.01^b^	1.28 × 10^−7^ ± 0.04^a^	2.26 ± 0.20^c^	59.43 ± 1.60^a^
9	5.5 (0)	15 (−1)	0.000 (−1)	0.03 ± 0.00^b^	5.56 × 10^−8^ ± 0.04^a^	1.36 ± 0.54^a^	62.52 ± 0.09^a^
10	5.5 (0)	25 (1)	0.000 (−1)	0.03 ± 0.01^b^	8.34 × 10^−8^ ± 0.81^a^	0.83 ± 0.04^a^	64.91 ± 0.91^a^
11	5.5 (0)	15 (−1)	0.750 (1)	0.02 ± 0.01^a^	7.31 × 10^−8^ ± 0.34^a^	2.09 ± 0.27^c^	67.84 ± 0.74^a^
12	5.5 (0)	25 (1)	0.750 (1)	0.03 ± 0.00^b^	9.50 × 10^−8^ ± 0.33^a^	0.68 ± 0.16^a^	61.91 ± 0.83^a^
13	5.5 (0)	20 (0)	0.375 (0)	0.02 ± 0.00^a^	6.74 × 10^−8^ ± 0.42^a^	1.34 ± 0.02^a^	59.90 ± 1.50^a^
14	5.5 (0)	20 (0)	0.375 (0)	0.02 ± 0.00^a^	6.90 × 10^−8^ ± 0.32^a^	0.93 ± 0.15^a^	59.94 ± 0.02^a^
15	5.5 (0)	20 (0)	0.375 (0)	0.02 ± 0.00^a^	6.79 × 10^−8^ ± 0.07^a^	1.18 ± 0.32^a^	59.91 ± 0.05^a^

Results show the mean ± SD. Results with the same letter in the column are not significantly different (*p* < 0.05) by the Tukey test. T, thickness; WVP, water vapor permeability; TS, tensile strength.

**TABLE 2:** ANOVA obtained for the model fitting.

Response	Source	Sum of squares	*df*	Mean square	*F*-value	*p*-value	Model Summary
T	Quadratic model	0.0004	9	0.0000	24.90	0.0012*	R^2^ = 0.97 Adj R^2^ = 0.93
	Residual	9.91 × 10^−6^	5	1.98 × 10^−6^	-	-	
	Lack of fit	9.25 × 10^−6^	3	3.08 × 10^−6^	9.25	0.0991	
	Pure error	6.66 × 10^−7^	2	3.33 × 10^−7^	-	-	
	Total	0.0005	14	-	-	-	
WVP	Reduced cubic model	1.55 × 10^−14^	11	1.41 × 10^−15^	12.82	0.0294*	R^2^ = 0.97 Adj R^2^ = 0.90
	Residual	3.30 × 10^−16^	3	1.10 × 10^−16^	-	-	
	Lack of fit	3.29 × 10^−16^	1	3.29 × 10^−16^	503.51	0.0020*	
	Pure error	1.30 × 10^−18^	2	6.53 × 10^−19^	-	-	
	Total	1.58 × 10^−14^	14	-	-	-	
TS	Quadratic model	8.18	9	0.9084	14.09	0.0047*	R^2^ = 0.96 Adj R^2^ = 0.89
	Residual	0.3223	5	0.0645	-	-	
	Lack of fit	0.3095	3	0.1032	16.12	0.0590	
	Pure error	0.0128	2	0.0064	-	-	
	Total	8.50	14	-	-	-	
DPPH radical scavenging activity	Reduced cubic model	100.53	11	9.14	10.36	0.0396*	R^2^ = 0.99 Adj R^2^ = 0.88
	Residual	2.65	3	0.8820	-	-	
	Lack of fit	2.65	1	2.65	6,103.8	0.0002*	
	Pure error	0.0009	2	0.0004	-	-	
	Total	103.17	14	-	-	-	

*Significant at 5% level. T, thickness; WVP, water vapor permeability; TS, tensile strength.

**TABLE 3:** Regression coefficients for response variables in the experimental design.

Regression coefficients	T	WVP	TS	DPPH radical Scavenging activity
Constant	0.089824	−7.69 × 10^−8^	−3.79403	−13.13704
$x_{1}$	−0.010565*	6.21 × 10^−8^*	0.729722*	19.82741*
$x_{2}$	−0.004183*	1.37 × 10^−8^	0.342917*	8.25300
$x_{3}$	−0.022111*	−1.45 × 10^−7^	−0.471111	−56.82519
$x_{1}^{2}$	0.000741	−7.15 × 10^−9^*	0.100556	−0.070370*
$x_{2}^{2}$	0.000057	9.23 × 10^−11^	0.001050	−0.195733*
$x_{3}^{2}$	0.033185*	4.50 × 10^−8^	−0.328889	13.52296*
$x_{1}x_{2}$	0.000667*	−7.29 × 10^−9^	−0.085667*	−2.15667
$x_{1}x_{3}$	−0.004444*	5.01 × 10^−8^	0.604444*	22.05926*
$x_{2}x_{3}$	0.000667	−7.86 × 10^−10^	−0.117333	1.10933*
$x_{1}^{2}x_{2}$	-	8.42 × 10^−10^	-	-
$x_{1}^{2}x_{3}$	-	−4.26 × 10^−9^	-	−1.68296
$x_{1\;}x_{2}^{2}$	-	-	-	0.053600

*Significant at 5% level. T, thickness; WVP, water vapor permeability; TS, tensile strength.

### 3.3 Structural analysis of the films

**FIGURE 2:**
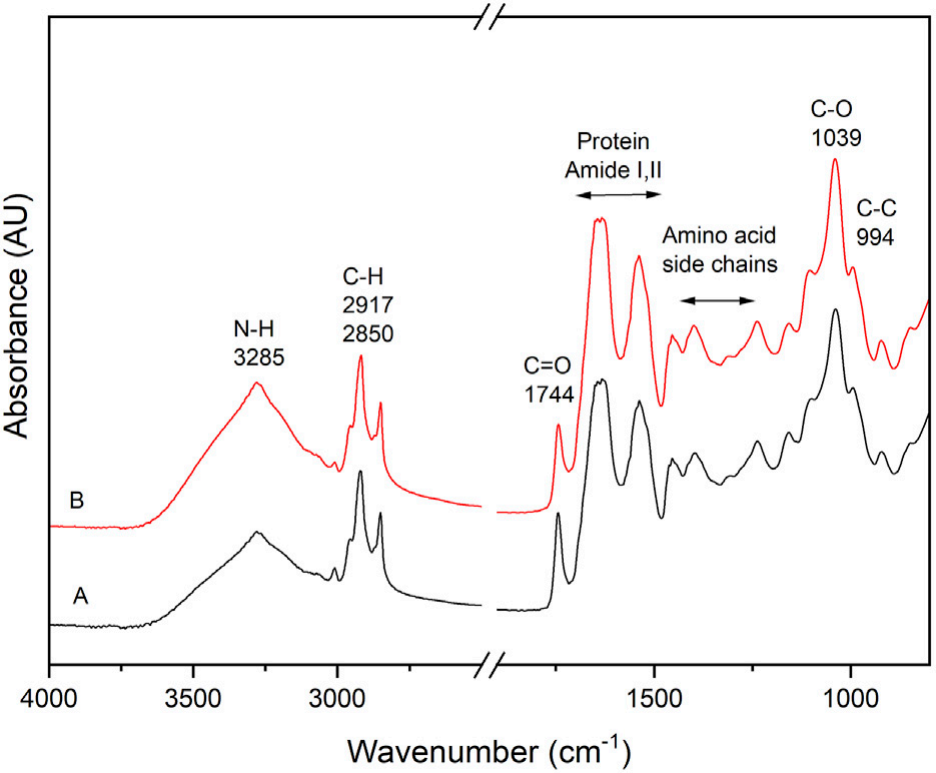
FTIR spectrum of the film with **(A)**: 0% nanoparticles, 7% chia flour, 20% glycerol, and **(B)**: nanocomposite film with 0.75% nanoparticles, 7% chia flour and 20% glycerol.

Figure 2 shows the FTIR spectra of the films prepared without (Figure 2A) and with nanoparticles (Figure 2B). In both spectra, bands at 3,285 cm^−1^ related to the N-H vibrations of the proteins were observed. The bands present at 2,917 and 2,850 cm^-1^ correspond to the vibrations of the C-H bond of proteins, carbohydrates, and glycerol. On the other hand, the signals at 1744 cm^−1^ arise from the stretching vibration of the C=O group of the lipids. The bands related to amides I and II of the proteins were observed at 1,641 and 1,537 cm^−1^ (Wang et al., 2020). Signals from the methyl methylene groups present in lipids and proteins were observed at 1,452 cm^−^. Finally, the bands located at 1,039 and 994 cm^−1^ are due to the C–O and C–C vibrations of the plasticizing agent. No differences were observed between the spectra of the films without nanoparticles and for the nanocomposite films. The concentration of the nanoparticles is so low that their presence does not cause spectral changes.

### 3.4 Analysis of response surface

**FIGURE 3:**
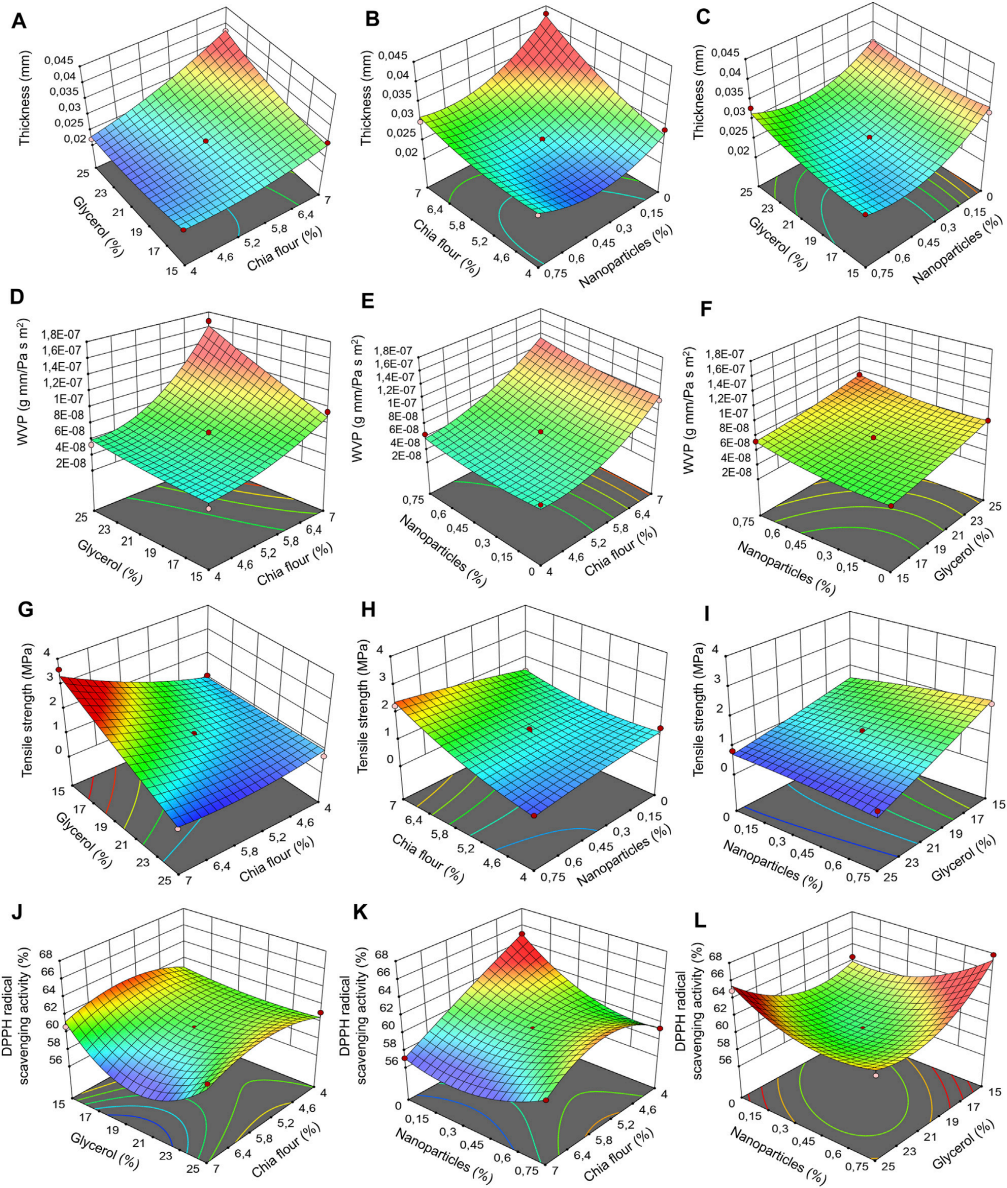
Response surface plots of the combined effects of independent variables of the simple and nanocomposite chia flour films with chitosan particles loaded with chia extract (ChCNp). **(A–C)** thickness, **(D–F)** water vapor permeability (WVP), **(G–I)** tensile strength, and **(J–L**) antioxidant activity.

Three-dimensional response surface graphs were constructed between two independent variables, keeping the third variable constant (Figure 3). The behaviours found in each of the study responses are described below.

#### 3.4.1 Physical properties

The films without nanoparticles were brown in color and homogeneous (Figure 1B). On the other hand, nanocomposite chia flour films were heterogeneous, flexible, and rough, due to the incorporation of the particles (Figure 1C). Thickness values of the chia flour nanocomposite films range from 0.02 to 0.04 mm (Table 1). The T was 10 times less than that of films of chia flour mixed with corn starch (Dick et al., 2015a) and similar to that of mucilage films without particles and mucilage films with cellulose nanofibers and starch nanocrystals (Dick et al., 2015b; Mujtaba et al., 2019a,b). Statistical analysis showed that chia flour, glycerol, and ChCNp had a significant impact on this parameter (Table 3) (Figures 3A–C). The addition of the nanoparticles generated a decrease in the T of the films (Figure 3C). The results are similar to those for mucilage films with starch nanocrystals (Mujtaba et al., 2019b). According to this research, the decrease can be attributed to the favorable interaction between the mucilage and the nanoparticles. Cabedo and Gamez-Pérez (2018) described that the nanoparticles in the films can be dispersed, intercalated, or aggregated, or there can be a combination of these. The intercalated particles generate a decrease in the thickness of the films according to Shariatinia et al. (2017). The final equation in terms of real variables for T is the following Equation 7:

$$\begin{array}{l} Y_{T}=0.089824-0.010565flour-0.004183glycerol-0.022111particles+0.000741{flour}^{2}+0.000057{glycerol}^{2}+0.033185{particles}^{2}\\ +0.000667flour*glycerol-0.004444flour*particles-0.000667glycerol*particles \end{array}$$ (7)

#### 3.4.2 Barrier properties

The WVP of the films was 3.89 × 10^−8^–1.68 × 10^−7^ g mm/Pa s m^2^. These values were similar to those reported in chia flour and mucilage films (Dick et al., 2015a,b), but better than the WVP for the mucilage chia films combined with concentrated whey protein (Muñoz et al., 2012). The WVP of the nanocomposite chia flour films were higher than those reported for synthetic films such as polypropylene (PP) (4.7 × 10^−9^ g mm/Pa s m^2^) and polyethylene terephthalate (PET) (1.3 × 10^−9^ g mm/Pa s m^2^) (Janjarasskul and Krochta, 2010). The WVP was significantly affected by the chia flour content (Table 3). The WVP increased with the concentration of chia flour (Figures 3D–F). Chia seed flour contains mainly carbohydrates and proteins. Mucilage is made up of arabinose, xylose, glucose, glucuronic acid, galactose, and galacturonic acid (Beikzadeh et al., 2020) and has a high water-retention capacity (Urbizo-Reyes et al., 2020), so increasing its concentration in the film can favor the WVP. The ChCNp did not significantly influence the WVP of chia flour films. Comparable results have been reported by other authors with some concentrations of nanoparticles (Ju et al., 2020; Roy and Rhim, 2021). Possibly, the concentration of the particles used is low, so its effect on this property is not appreciated. In this work, the possibility of adding more nanoparticles was not considered because the film formed was very heterogeneous. The final equation in terms of real variables for the WVP is the Equation 8:

$$\begin{array}{l} Y_{WVP}=-7.69\;x10^{-8}+6.21x10^{-8}\;flour+1.37\;x10^{-8}glycerol-1.45\;x10^{-7}particles-7.15\;x10^{-9}{flour}^{2}+9.23x10^{-11}{glycerol}^{2}\\ +4.50x10^{-8}{particles}^{2}-7.29\;x10^{-9}flour*glycerol+5.01x10^{-8}flour*particles\\ -7.86\;x10^{-10}glycerol*particles+8.42\;x10^{-10}{flour}^{2}*glycerol-4{.26x10}^{-9}{flour}^{2}*particles \end{array}$$ (8)

#### 3.4.3 Mechanical properties

The TS of the films was 0.67–3.59 MPa. These values are comparable to those reported for chia flour films (0.77 MPa) and chia mucilage films mixed with whey protein (3.79 MPa) (Muñoz et al., 2012; Dick et al., 2015b). The TS was significantly influenced by the flour and glycerol content, as well as by the interactions between flour and glycerol and flour and nanoparticles (*p* < 0.05) (Table 3). The films with a higher concentration of chia flour presented a higher TS (Figure 3G). When the polymer concentration is increased, more interactions occur between them, increasing the resistance of the films. Glycerol negatively affected this parameter: its increase caused a decrease in TS (Figures 3G,I). These results agree with those reported by Urbizo-Reyes et al. (2020). The hydroxyl groups of glycerol contribute to the incorporation of water molecules, which can increase the molecular mobility of the films, decreasing their resistance. On the other hand, when the concentrations of flour and nanoparticles were simultaneously increased, the films presented a higher TS (Figure 3H). The greatest reinforcement was obtained with concentrations of chia flour of 6.4%–7% and 0.45%–0.75% of nanoparticles. The nanoparticles interacted best with chia flour at high concentrations. The main interactions that have been described between chitosan nanoparticles and polymeric matrices are intermolecular interactions due to hydrogen bonds (Zhao et al., 2023). Although the nanoparticles agglomerated (Figure 1C), they managed to reinforce the films. These results are similar to those reported by Shapi’i et al. (2022) for starch films incorporating chitosan nanoparticles and by Hosseini et al. (2015) for fish gelatine films reinforced with chitosan nanoparticles. The final equation in terms of real variables for TS is the Equation 9:

$$\begin{array}{l} Y_{TS}=-3.79403+0.729722flour+0.342917glycerol-0.471111particles+0.100556{flour}^{2}+0.001050{glycerol}^{2}-0.328889{particles}^{2}\\ -0.085667flour*glycerol+0.604444flour*particles-0.117333glycerol*particles \end{array}$$ (9)

#### 3.4.4 Antioxidant properties

The films had a DPPH scavenging activity of 57.12%–67.84%. The antioxidant capacity found was greater than that reported in chitosan films added with chitosan particles loaded with quercetin (Roy and Rhim, 2021). This response was significantly affected by the flour content and by the interactions between flour, glycerol, and nanoparticles (Table 3). The highest antioxidant activity was found in the films made in the range of 4.6%–6.4% chia flour (Figure 3J). As described previously, phenolic compounds such as myricetin, quercetin, and kaempferol have been identified in chia flour and its ethanolic extracts, which can stabilize free radicals (Alcântara et al., 2019). On the other hand, it was observed that the antioxidant activity increases from 60% to 68% at low concentrations of glycerol and high concentrations of ChCNp (Figure 3L). ChCNp showed a positive effect on the antioxidant activity of the films. Similar results were reported by other authors in films added with nanoparticles loaded with antioxidants (Zhang and Zhao, 2017; Ezati and Rhim, 2021; Roy and Rhim, 2021). In previous studies carried out in the working group, it was found that the synthesized nanoparticles have an antioxidant capacity when they are subjected to 40°C (drying temperature of the films) and at pHs of 6 and 10 (Morales-Olán et al., 2021) by which the antioxidant capacity of these films could be increased by having contact with foods with these pHs. On the other hand, it is suggested to evaluate the antioxidant capacity of the films during their storage. The final equation in terms of real variables is the Equation 10:

$$\begin{array}{l} Y_{DPPH}=-13.13704+19.82741flour+8.25300glycerol-56.82519particles-0.070370{flour}^{2}-0.195733{glycerol}^{2}+13.52296{particles}^{2}\\ -2.15667flour*glycerol+22.05926flour*particles-1.10933glycerol*particles\\ -1.68296{flour}^{2}*particles+0.053600flour*{glycerol}^{2} \end{array}$$ (10)

## 4 Conclusion

In this study, the influence of the content of flour, glycerol and chitosan nanoparticles loaded with antioxidants on the physical, barrier, mechanical, structural and antioxidant properties of chia flour films was evaluated. The variables studied generated different effects on the properties of the films. Increasing the content of chia flour negatively affected the WVP; however, it improved the TS and antioxidant capacity of the films. On the other hand, the increase in glycerol concentration generated brittle films, due to its hydrophilic behavior and plasticizing capacity. The addition of nanoparticles produced heterogeneous films; however, the nanoparticles showed a significant effect on the thickness and improved the mechanical and antioxidant properties of the films. The results demonstrate that chitosan nanoparticles loaded with antioxidants improve the properties of chia flour films and allow the generation of nanocomposite films with antioxidant capacity. These films could be used as an active packaging alternative for food preservation.

## Data Availability

The raw data supporting the conclusions of this article will be made available by the authors, without undue reservation.

## Abbreviations

ChCNp, Chia extract-loaded chitosan nanoparticles; EE, Encapsulation efficiency; LC, Loading capacity; WVP, Water vapor permeability; TS, Tensile strength.
